# Evaluation of informal payments to health care professionals and the influential factors in Urmia city hospitals, Iran

**Published:** 2018-07-11

**Authors:** Abdolvahed Khodamoradi, Arash Rashidian, Reza Daryabeygi‐Khotbehsara, Siamak Aghlmand

**Affiliations:** 1 *Researcher, Department of Health Economics, Social Security Research Institute, Tehran, Iran.*; 2 *Professor, Department of Health Management and Economics, School of Public Health, Tehran University of Medical Sciences, Tehran, Iran. *; 3 *Department of Clinical Nutrition, School of Nutritional Sciences and Dietetics, Tehran University of Medical Sciences, Tehran, Iran.*; 4 *Associate Professor, Department of Public Health, School of Public Health, Urmia University of Medical Sciences, Urmia, Iran.*

**Keywords:** *Out-of-pocket payment*, *Informal payment*, *Health care professionals*, *Iran*

## Abstract

Informal payments refer to sums that patients may pay to individual or organizational health care providers outside of the official payment channels or approved fee schedules. The aim of the current research was to investigate informal payments and related influential factors in Urmia city hospitals.

The present study was a cross-sectional survey conducted among post-discharged patients from all Urmia city hospitals during one Iranian calendar month (January 21 to March 19, 2013). Simple random sampling was used to recruit 265 patients to undergo assessment via phone call interviews and complete a questionnaire. Data analysis was performed using SPSS software for descriptive reports, and EViews software for determination of factors affecting informal payments.

Eleven percent of the patients had made informal payments to physicians (mean amount: 503,000 Tomans, equivalent of $412), 5% to nurses (mean amount: 20,000 Tomans, equivalent of $16), and 17% to other employees (mean amount: 16,000 Tomans, equivalent of $13). Hospital ownership, patients’ place of residence, education and income significantly influenced the payments. Most substantially, patients receiving surgical care were 100 times more likely to make informal payments compared to those who had received non-surgical inpatient care.

The present study showed that although informal payment is illegal in Iran, it is a common practice among hospitalized patients, and has now become a challenge for the health system. Considering the high prevalence of informal payments and their severe impacts on equity and justice, policymakers have focused on this phenomenon to reduce and eliminate it.

## Introduction

Many countries around the world have suffered the consequences of informal payments for health services, which is one of the important issues in health care systems. The extent and importance of informal payments for health care services have been discussed in various sectors, especially after the reform attempts of the former Soviet Union and increased focus on the health care systems of economies facing transition (1).

Informal payments are made to individual or organizational health care providers by patients outside of the official payment channels (2), and may take various forms. Instances include cash, kind (goods and services such as drugs, food, nursing care, blood supply, laboratory tests), and gifts (chocolate, juice, cookies, flowers) (3 - 5). From an economic perspective, informal payments fall in the category of out-of-pocket (OOP) payments, as they have the same effect on the financial burden for patients and their access to care (6). Due to their negative consequences, informal payments have become a common policy concern in low-, middle-, and occasionally high-income countries since three decades ago (5).

There are various reasons and causes for informal payments, including: a) the culture of gift giving in certain countries; b) the low income of medical staff; c) the lack of resources and supplies in provider organizations where an informal payment might result in better care; d) the bargaining power of the medical staff with the patients; e) inadequate supervision of the health system; and h) a shortage of regulations and law enforcement (7 - 9).

Informal payments may have many negative consequences, including decreased access to and utilization of care; increased inequity in care provision and instigating a sense of hopelessness among the poor; decreased motivation for delivering quality services at authorized costs among health care providers; increased corruption in the health sector resulting from secrecy and unhealthy financial relationships; provision of incorrect information about the costs of patients’ illnesses; and an increase in the patients’ share of costs. Finally, informal payments may provide false information about real costs, which may lead to incorrect decisions and policies resulting in impaired performances and hinder the required reforms in health systems (1, 10, 11). On the other hand, some authors have suggested that a portion of informal payments can be regarded as the participation fee to ensure that the employees receiving the payments will remain in their workplace and continue to provide care (12).

Informal payments vary largely in different countries: 55, 81 and 96 percent of users in Cambodia, Vietnam and Pakistan incurred informal payments, respectively. Lower levels of such payments have been reported in other countries, for instance in Thailand and Peru it affected less than three percent of the users. Similarly, low or high levels of informal payments have been reported in other regions (2). Furthermore, the prevalence of informal payment is higher in inpatient (hospital) services than outpatient (13).

Iran is a higher middle-income economy with a population of over 80 million. Total expenditure on health is estimated to be 6.2% of Gross Domestic Product (GDP) in 2008 (based on World Bank; 7.1% in 2014) (14). The government and private section’s proportion of health expenditures are 55.1% and 44.9%, respectively. Ninety-five percent of private section’s health expenditures are out-of-pocket (15), although the majority of the population is covered by health care insurance funds, estimated at 83% of the population in 2010 (16), and over 90% presently. It has been argued that a former policy of “hospital autonomy” across the public sector in Iran might have resulted in a transfer of a portion of the health care costs to users. Limited evidence from the city of Tehran suggests that the rate of informal payments to physicians in public hospitals was close to zero, but these studies did not include the private sector, or public hospitals not affiliated with the Ministry of Health (MOH) entities (e.g. Social Security Organization hospitals (SSOH)) (17). 

Informal payments increase out-of-pocket payments; in addition, they may exacerbate health catastrophic expenditures and poverty among the poor, and have negative effects on equity and health outcomes. Considering the scarcity of accurate information about the extent of informal payments and limitations of comprehensive studies in this field in Iran, we decided to conduct a study on the issue. We set out to estimate the frequency and extent of informal payments and their determinants so that the information could be used in policymaking geared toward reducing informal payments and out-of-pocket expenditures. 

In recent years, some studies have been performed on informal payments in Iran. A number of these studies have assessed physicians’ attitudes and experiences toward informal payment (18), and some have targeted teaching hospitals affiliated with the Ministry of Health (17, 19). Moreover, some other studies have assessed influential factors, reasons and outcomes of informal payment (20 - 24). The current study is different from the research mentioned above in that it has assessed the issue in non-teaching hospitals (including private and social security hospitals). Additional strength to the present study is the investigation of informal payment through patients’ viewpoint, which is eventually assessed for influential factors by an econometric model.

In this study, we measured informal payments according to frequency, mean, type, nature, and the patients’ motivation in making these payments to three groups of medical staff (physicians, nurses, and other staff), in three different types of hospital ownerships: MOH teaching hospitals (affiliated with universities of medical sciences and health services at the provincial level under the supervision of the Ministry of Health and Medical Education); non-MOH public hospitals (affiliated with governmental organizations other than the Ministry of Health and Medical Education, such as the Social Security Organization or Petroleum Industry Health Organization); and private hospitals. We also identified factors influencing informal payments to physicians. 

## Method

In this cross-sectional study, we surveyed informal payments across all inpatient admissions to the hospitals located in Urmia, the capital of West Azerbaijan Province in the northwest of Iran. The province shares borders with Azerbaijan, Iraq and Turkey. According to the 2012 census, Urmia had a population of 1,265,721 (700,000 households), and the population of the whole province was 3,080,576 in the same year. The hospitals in Urmia offer specialized secondary and tertiary care services, serving the West Azerbaijan Province, and occasionally the neighboring countries. 

The study population included all patients who were discharged in one Iranian calendar month (Jan 21 to March 19, 2013) from Urmia hospitals. Hospitals that were eligible for inclusion in the study included: five MOH teaching hospitals affiliated with the Urmia University of Medical Sciences, one hospital affiliated with the Social Security Organization (public non-MOH), and three private hospitals, with a total number of 1727 beds. 

The calculations indicated that a sample size of 265 would suffice. In total, 10791 patients were discharged from the hospitals during the study period. As the first step in sample selection, the list of all the patients discharged during the specified period were obtained from the hospitals and collated into one sampling frame, and were subsequently numbered from 1 to 10791. Considering the possibility of missing samples and non-responses, 500 discharged patients were selected using a random number table and the simple random sampling approach.

In the next step, the list of the 500 patients was ordered so that the first person on the sample list was the first person selected through the random number table; and the last person on the list was the last (500^th^) patient selected through the random number table. 

For data collection, we contacted patients starting at the top of this list and continued until the required sample size of 265 was obtained. The target sample size was reached after contacting the 332^nd^ patient on the list, giving us a response rate of about 80%. 

The data were collected via discharge lists and telephone conversations with the patients or their informed families. The data collection tool contained 40 questions in five sections: the first section contained questions about provider and hospital care characteristics, total out-of-pocket payment to the hospital, and whether any informal payment had occurred. The following three sections focused on the details of informal payments, if any, to physicians, nurses and other hospital staff. The last section consisted of the socio-economic and demographic characteristics of the patients. 

A preliminary tool was prepared using a review of the literature. Next, each member of the research team gave feedback on the tool in terms of its completeness and face validity. The tool was finalized after group discussions among the research team. 

Based on consensus across literature, the current definition of informal payment covers various forms such as cash, kind and gift, each reported separately. However, in Iranian culture, informal payments specifically refer to cash, whereas gifts are usually a means of acknowledgement (22).

Hospital professions were divided into three groups: physicians, nurses and other staff (e.g. office workers, housekeeping crew, watchmen, midwifes and other clinical staff).

Data were included in a data sheet and were analyzed using univariate (e.g. mean and frequency) and multivariate (i.e. logistic regression) analytical techniques. The latter was conducted to identify influential factors on informal payments to physicians, and the significance level was set at 0.05. The model was specified as: Logit Y = B0 + B1X1 + B2X2 + ... + BnXn. Y was a dummy variable, so that Y = 1 if any informal payment had occurred and, Y = 0 if no informal payments had been made. We used the Hosmer and Lemeshow statistics and Wald test, which indicated adequacy of goodness of fit for the models. 

The study was approved by the ethics committee of Tehran University of Medical Sciences, Tehran, Iran, November 2013.

## Results

Of the 265 patients in this study, 149, 69, and 47 were hospitalized in teaching, private and social security hospital, respectively. Distribution of respondents is presented in table 1 as below.

**Table 1:** Descriptive statistics of respondents

**Variable**	**N**	**%**
*Gender* Female Male	149 116	56.2 43.8
*Patient residing in* Urmia (site of hospitals) Other towns or cities A village	132 57 76	49.8 21.5 28.7
*Household head education* Illiterate Primary school High School College/University Graduate	71 114 48 32	26.8 43 18.1 12.1
*Income* I (the lowest) II III IV V VI (the highest) Not clear	30 62 70 40 23 26 14	11.3 23.4 26.4 15.1 8.7 9.8 5.3
*Hospital type* Teaching Private Social	149 69 47	56.2 26.1 17.7
*Treatment type* Surgery Medical therapy (excluding surgery) Natural childbirth Caesarean operation	118 111 14 22	44.5 41.9 5.3 8.3

*Frequency, Size and Nature of Informal Payments*

Of the total 265 respondents in the study sample, 79 (29.8%) of the patients reported informal (cash or non-cash) payments to hospital staff. This figure varied depending on the type of hospital; for example, in private and social security hospitals, informal payments were more than twice the amount reported in teaching hospitals. Among these, 30 (11.3%) patients made informal payments to physicians (mean amount: 503,000 Tomans, equivalent of $412), 14 (5%) to nurses (mean amount: 20,000 Tomans, equivalent of $16), and 45 (16.9%) to other hospital staff (mean amount: 16,000 Tomans, equivalent of $13). The amount of cash paid informally to non-physicians was insignificant, and some patients had made informal payments to two or three groups of staff in one hospital admission.

Informal payments to physicians, nurses and other staff also varied depending on the type of hospital. For example, 34% of the patients in the social security hospitals and 15% of the patients in the private hospitals reported informal payments to physicians, but only 2% of the patients admitted to teaching hospitals reported informal payments to physicians (Table 2).

**Table 2:** Frequency, percentage and mean amounts of informal payments by hospital type

**Hospital ** **type**	**No. of ** **patients**	**Mean out-of-pocket ** **payments** * ** (CI) $**	**Mean informal ** **payments (CI) $**	**Proportion of patients who made informal payments to**			
				**physicians**	**nurses**	**other staff**	**All**
Teaching	149	128 (106-181)	7 (1-13)	3 (2%)	9 (6%)	22 (14.7%)	29 (19.4%)
Private	69	785 (602-1096)	71 (13-129)	11 (16%)	5 (7%)	17 (24.6%)	29 (42%)
Social ecurity	47	230 (115-363)	153 (52-254)	16 (34%)	0	6 (12.7%)	21 (44.6%)
Total	265	317 (265-423)	50 (25-74)	30 (11.3%)	14 (5%)	45 (16.9%)	79 (29.8%)

*Exchange rate (April, 2013): 1$ = 1220 Tomans*

* *including informal payments*

The findings indicated that informal payments comprised 15.5% of the total out-of-pocket expenses that patients had to meet, which was $49 (CI: 24 - 71) out of $317 (CI: 241 - 393) for each patient. The amount of informal payments in the social security hospital was considerably higher than those made to physicians in the private or teaching hospitals (two and 22 times higher, respectively).

In this study, the largest part of informal payments had been made in cash. We classified informal payments in two categories of cash and non-cash payments. The results showed that 67% of the payments were in cash, while 28% were non-cash, and 5% were both cash and non-cash. Most non-cash payments (90%) consisted of flowers and gifts, and 10% were in the form of goods. This was true for all types of hospitals, and the total amount of cash payments was higher than non-cash ones. In terms of personnel, payments to physicians (87%) and to other staff (64%) were mainly in cash, while payments to nurses (64%) were mostly non-cash.

We also assessed mandatory payments (requested by staff) and voluntary payments (offered by patients) and found that 70% of the payments were voluntary and 30% were mandatory. It should be mentioned that more than 90% of the payments to nurses and other staff were voluntary, but 83% of the payments to physicians were mandatory and had been requested by physicians as extra payment.

Payments to physicians were all in cash and requested by the physicians. Most (80%) of these payments were made before hospital admission, 16.7% were made in the hospital, and an additional 3.3% occurred after discharge. In the case of nurses and other staff, the payments were all made in the hospitals.

The respondents were asked to choose the “best” response among the choices shown in table 3. In the majority of the cases (73.4%), the patients made the payment at the request of the physician. Acknowledging the physician’s efforts or receiving high-quality services (the fear that if they did not pay, they would not receive appropriate services) were mentioned as other reasons for payments. The scenario was different for nurses and other staff, in that the majority of the payments (71.4% and 64.4 respectively) were made to acknowledge the efforts or show gratitude, or as gifts and presents (Table 3). 

**Table 3:** Reasons for informal payments to physicians and nurses

**Reasons for informal payments**	**Physician**	**Nurse**	**Other staff**
Acknowledgement, gratitude, gifts and presents	4 (13.3%)	10 (71.4%)	29 (64.4%)
High-quality service (without provider request)	4 (13.3%)	3 (21.4%)	12 (26.6%)
Provider request	22 (73.4%)	1 (7.2%)	4 (8.8%)

We also identified the type of personnel (for staff other than physicians and nurses) who received informal payments. Most of the payments were made to the housekeeping crew (71.1%) followed by watchmen (2.2%). The rest of the payments were made to a group of personnel or a ward (such as labor or pediatric ward).

*Factors Influencing Informal Payments*

Among physicians, general surgeons received the most informal payments at 42%, followed by ophthalmologists, otorhinolaryngologists, and maxillofacial surgeons (24%), internists, neurologists, and hemato-oncologists (10%), orthopedic surgeons (7.1%), urologists (6.2%), and obstetricians and gynecologists (5.5%). Cardiologists and pediatricians received no informal payments. However, in general, the logistic regression model showed no significant correlation between the physician’s specialty and probability of receiving informal payments. Moreover, the percentage of informal payments to physicians was 20% in surgical patients (29 out of 140 persons) and 0.8% in non-surgical patients (1 out of 125 persons). The current study model showed that surgery had a significant positive effect on the occurrence of informal payments (P-value = 0.00). 

Informal payments to nurses and other staff were negligible in size and mostly in the form of gifts to show gratitude, but in the case of physicians, they were mandatory and in most cases much higher than their official fees. Therefore, we assessed the factors that influence informal payments to physicians. The results are shown in table 4 below.

**Table 4:** The results of the logistic regression model (Logit)

**Variable**	**Coefficient**	**Marginal Effect**	**Std. Error**	**Z-Statistic**	**Prob.**
*Hospital type* Teaching Private Social Security	1 0.92 1.43	- 2.50 4.18	- 0.47 0.35	- 1.94 4.05	- 0.05 0.00
Supplemental insurance (Yes, No)	- 0.65	0.52	1.09	- 0.59	0.55
*Patient residing in* Urmia Other towns or cities A village	1 3.04 1.86	- 20.84 6.42	- 0.85 1.00	- 3.56 1.85	- 0.00 0.06
*Male gender*	0.44	1.56	0.72	0.61	0.54
Surgery (Yes, No)	4.65	103.11	1.44	- 3.21	0.00
*Physician specialties* General Surgeon Maxillofacial surgeons Hemato-Oncologists Nephrology or Urology Orthopedic Surgeon Obstetrics and Gynecologist	1 0.92 1.51 - 2.23 - 2.21 - 1.47	- 2.52 4.49 0.11 0.11 0.23	- 0.86 1.15 2.50 1.48 1.05	- 1.06 1.30 - 0.89 - 1.49 - 1.40	- 0.28 0.19 0.37 0.13 0.16
*Household head education* Illiterate Primary school High School College/University Graduate	1 3.86 3.93 3.77	- 47.51 51.06 43.24	- 1.47 1.52 1.66	- 2.62 2.58 2.26	- 0.00 0.00 0.02
*Income*	0.16	1.18	0.07	2.25	0.02
C	- 8.98	-	2.08	- 4.30	0.00

*Goodness of fit of the model: H-L Value: 14.30, P-value: 0.07; Wald’s Chi-squared: 8.06, P-value: < 0.01; McFadden R-squared: 0.60*

Logistic regression showed that the probability of informal payments to physician was 4.1 times higher in patients hospitalized in the social security hospital compared to teaching hospitals. Additionally, informal payments were more likely to have occurred in the private hospitals than the teaching hospitals, but the difference was not statistically significant. We could also find no significant relationship between the occurrence of informal payments and the variables of complementary medical insurance, specialty of the physician, and the patient’s gender.

Logistic regression also showed that the probability of informal payments was 20 times higher if the patient’s place of residence was different from the city in which the hospital was located. Moreover, the occurrence of informal payments to surgeons was on average 103 times more than physicians in non-surgical roles. In other words, surgery increased the probability of informal payments by over 100 times. Another factor that positively affected informal payments was literacy of the household head. The logistic model also demonstrated that a higher income increased the probability of informal payments (Table 4).

## Discussion

This was one of the few studies that have assessed the potential impact of hospital ownership (e.g. public, private, etc.) on informal payments in hospitals in low- and middle-income countries. Our findings indicate that 30% of the hospitalized patients in any type of hospital had made informal payments to various members of staff, and 11% had made informal payments to physicians. Patients admitted to teaching hospitals were substantially less likely to make informal payments to physicians as compared with those admitted to social security and private hospitals. The present study findings were similar to the results of studies from Greece and pre-reform Turkey, where informal payment rates were 36% and 31%, respectively (5, 25).

Lewis reported an increase in informal payments from 3 to 96 percent worldwide (2). They also revealed informal payments to be higher in the inpatient sector compared to outpatient. Results from Stepurko et al. showed that prevalence of informal payment ranges from less than 10% to more than 70% (26). This wide range of payments may be prominently influenced by methods of data collection. Gaal et al. have discussed the underlying reasons for informal payments and provided four issues that account for diversity of the reported informal payments: a) sampling selection bias; b) incorrect classification of health care costs; c) inability to identify informal payments; and d) recall period (10). In addition, Lewis believes that data sources are varied, studies are undertaken in different area levels (regional, municipal, state or national), conducted on diverse populations (households, health care providers, or health centers), or pursue corruption (2). 

A published study in Iran assessed informal payments in teaching hospitals only in Tehran and the authors observed no informal payments to the physicians (17). This was in line with the current study results in which only 2% of the patients admitted to teaching hospitals had made informal payments to physicians. In a study by Vafaei Najar et al. conducted in two educational hospitals in Mashhad, results revealed that the prevalence of informal payment was approximately 6% and mainly for acknowledgement purposes to health care providers (19). Also, Parsa et al. demonstrated that acceptance of informal payments was higher among physicians working in private sector compared to the public sector (18), which is in a similar vein to current results.

It should be mentioned that informal payments to physicians were different in type and magnitude from payments to nurses and other staff. The physicians (specifically those providing surgical care) received payments that had higher amounts, were mandatory, and were more likely to be made in response to the direct requests of the patients, whereas 93% of the payments to nurses and 98% to other staff were voluntary. These findings showed that physicians were more likely to ask for informal payments, even though their formal earnings were higher than other members of the staff. The reason might be physicians’ higher income expectations that are not satisfied via their formal earnings (27), and may be further assessed in future studies. A study in Greece showed that of the 36% informal payments to physicians, 19% were mandatory and at the physicians’ request, and 17% were voluntary (25). 

We also observed that informal payments to physicians were more likely to occur in the social security hospital rather than teaching and private hospitals. This was interesting, since in Iran, social security hospitals have official zero co-payment policies, while teaching and private hospitals employ different rates of co-payment (28). As a result of the informal payments, patients were spending higher total out-of-pocket payments in social security hospitals than in the teaching hospitals. Such policies only focus on reducing user charges without adequate regulatory mechanisms and might result in lowered benefit to patients (1). We also found few studies that compared informal payments across hospitals with different ownerships. Liaropoulos et al. showed that the probability of informal payments to nurses was higher in private hospitals than governmental ones (25). Moreover, a study by Ozgen et al. in Turkey showed that the type of ownership of the service provider had a significant correlation with informal payments (5).

In the present study, the place of residence was significantly associated with informal payments. People from surrounding cities were more likely to make informal payments compared to those living in the city where the hospital was located. Congruent to our findings, studies conducted in Albania, Turkey, Serbia, Hungary, Greece and Russia found a positive association between the place of residence and informal payments (9, 29 - 33).

Moreover, informal payments were positively correlated with the educational level of the head of household. Illiterate patients made the lowest, and patients with academic education made the highest payments. One reason could be that people with higher education generally have larger incomes. In a similar vein, a number of studies (30 - 32, 34 - 36) identified the effect of education on informal payments. Furthermore, the probability of making informal payments was higher in households with larger incomes. A number of studies have also showed that household expenditure or income have a significant correlation with informal payments (5, 9, 31-38).

Informal payment does not exist or is barely seen in successful health systems throughout the world such as Great Britain, Denmark, Germany, Sweden, Netherland, Norway, Finland, Belgium, Portugal, Switzerland, France, Italy, Iceland, and so on (39). The present study showed, however, that in Iran, it is common practice among hospitalized patients and has now become a challenge for the health system. As a result and considering the high prevalence of informal payments and their severe impacts on equity and justice, policymakers have focused on this phenomenon to reduce and eliminate it. Since payments to physicians vary in different types of hospitals and different specialties, health managers should take measures that can address the problem of informal payment appropriately (40, 41). Health experts believe that informal payments are harmful to the health sector, the government, and the society, and should therefore be prevented. Some studies have proposed strategies to deal with the issue of informal payments. However, few studies have attempted to assess the effects of implementation programs aiming at elimination or reduction of informal payments, and have recommended policies against it instead. Examples of successful practical programs include universal health coverage, complementary private insurance, ratification of acts against informal payment, and promotion of public awareness via media (9, 36, 42). 

## Conclusion

With regard to specificities of the medical profession and the importance of public trust in physicians, it is essential to protect and reclaim the public faith in medical staff. Informal payment is among the most critical stimulators of public distrust that tarnishes the physician-patient relationship. In addition, the medical community is responsible for the well-being of the general public (including vulnerable populations) by ensuring that extra expenses are not imposed on people through under-the-table payments. Moreover, health system policymakers must outline felicitous strategies to reduce and eliminate informal payments. 

In this respect, experiences of other countries can be beneficial and should therefore be taken into consideration. Recognizing the structure and processing of health care systems of countries where informal payments are uncommon would be helpful for countries struggling with this issue, especially in terms of financial investment to eliminate class distinction. It should also be noted that there is no universal approach to elimination of informal payment, and formulation of a set of policies commensurate with the country’s condition is essential.
